# Commentary: The Incidence of Road Traffic Crashes Among Young People Aged 15–20 Years: Differences in Behavior, Lifestyle and Sociodemographic Indices in the Galilee and the Golan

**DOI:** 10.3389/fpubh.2021.651376

**Published:** 2021-03-04

**Authors:** Gerry Leisman, Igor Waksman

**Affiliations:** ^1^Faculty Social Welfare and Health Sciences, University of Haifa, Haifa, Israel; ^2^Neurofisiología Cliníca, Instituto de Neurología y Neurocirugía, Universidad de Ciencias Médicas, Havana, Cuba; ^3^Laboratory for Global Health, Department of Surgery B, Galilee Medical Center, Nahariya, Israel

**Keywords:** frontal lobes, adolescence, driving, accident, traumatic brain injury

## Introduction

Actuaries evaluate risk and opportunity by the application of statistical and financial analysis. They know that males under 26 and females under 23 are at significantly higher risk for traffic accidents (1) and yet they have no knowledge of frontal lobe development. Longitudinal neuroimaging studies demonstrate that the adolescent brain continues to mature well into the 20s (2). This has prompted intense interest in linking neuromaturation to maturity of judgment. Public policy is struggling to keep up with burgeoning interest in cognitive neuroscience and neuroimaging. According to the US Centers for Disease Control 27,000 individuals aged between 1 and 24 years die from poor decision-making (3–5). Besides murder and suicide, the majority of deaths are attributable to accidents and trauma (5). We know that the teenage years through the age of neurological maturity ~25 for males and 22 for females (6, 7), are an era in life highly associated with reckless behavior and decision making independently of the fact that teenagers are cognitively more mature than younger children (8). Surgical emergencies due to major and minor trauma testify to this – many of these injuries are preventable.

Actuaries have indicated, by offering higher insurance rates to this population, that reckless driving, including handling a vehicle under the influence of drugs or alcohol, is significantly more likely during this period of life relative to any other. The basis for most of these rash behaviors, while taken into account by insurance companies, does not seem to feature in public health policy for adolescents and young adults, mitigating risk behaviors or shaping sound decision making. These individuals are disproportionately prone to making bad and, oftentimes, fatal decisions, these being the greatest contributing factors to mortality in adolescents (5). The neurological contribution to this issue was not referred to in the paper to which this commentary is addressed, but this is worth studying. The determinants of health that the authors refer to: poverty; poor educational attainment; unemployment; and, low income have important correlations with brain development and executive brain function (7, 9). It is time that these social determinants are fully researched among the adolescents admitted with emergency surgery and trauma diagnoses.

**Figure 1:**
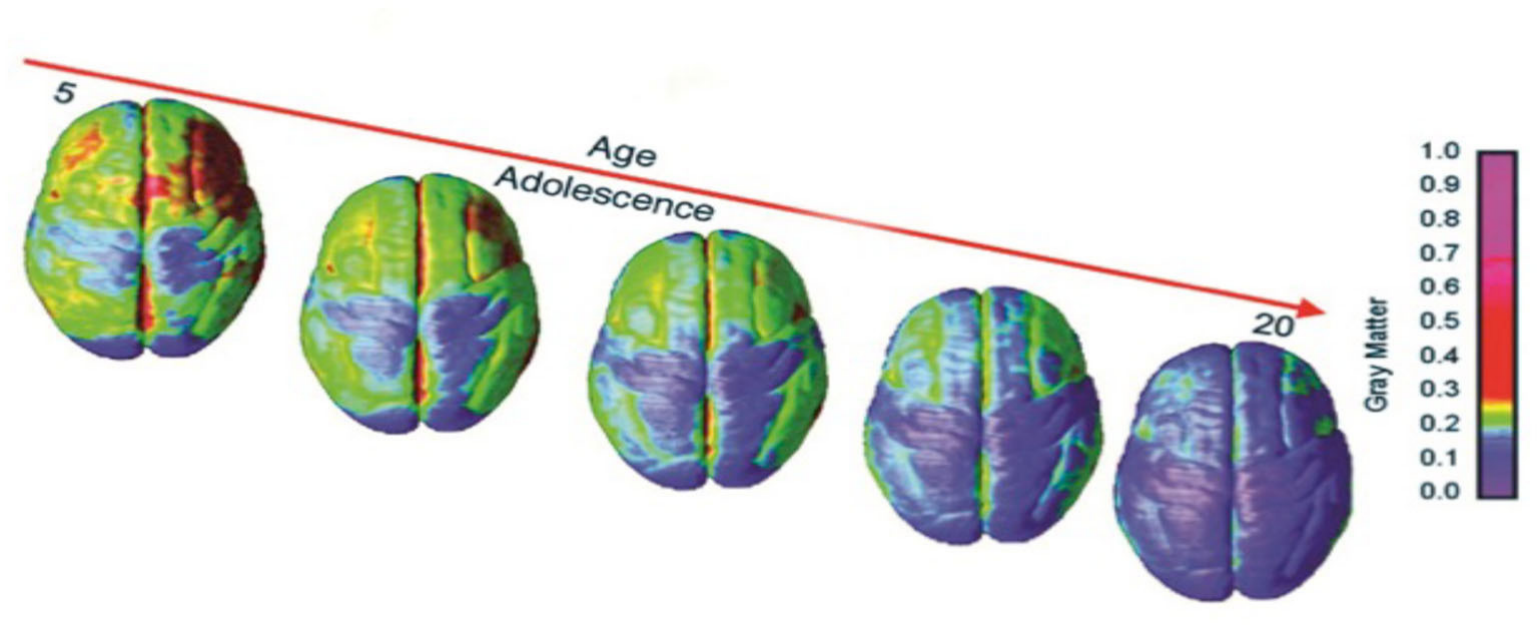
Right lateral and top views of the dynamic sequence of GM maturation over the cortical surface. The side bar shows a color representation in units of GM volume [modified from (23)].

Many have theorized why it is that teenagers might participate in unsafe behaviors. Perhaps, according to one view, maturational differences exist between cognitive and limbic control regions of the brain (10–12). While most adolescents somehow pass through the teenage years with few lifelong consequences, some do not (13, 14). We do not know whether we are observing forms of self-stimulatory behavior or immaturity of the connections subserving the control of impulsivity. What we do know, however, is that the literature supports the notion that sensation seeking is curvilinear in its development climaxing in the mid- to late teenage years (15). Impulse control, on the other hand, develops into adulthood in a linear fashion (15). The problem with the state of the current literature on the topic is that risk-taking is not simply a function of chronological age. We know from longitudinal fMRI studies (16) that gray matter volume tends to peak during early adolescence when gray matter decreases in volume which is related to synaptic pruning where we have learned that weaker connections are pruned so other circuits can increase and the brain's function can become more optimized for decision-making and executive function (17). We know that adolescent medial prefrontal cortex (mPFC) brain regions are more active during emotional response and that these areas employ different tactics in adolescence than in individuals at other stages of life. We know that the mPFC is one of the last areas of brain development to demonstrate indications of anatomical development. This development is not completed on average (i.e., maturity) until individuals reach their mid 20's (18–22). Figure 1 demonstrates the dynamic sequence of gray matter maturation over the cortical surface.

Pleasure and reward system function is less well-described as a consequence of development, but we have learned that activity in brain regions such as the nucleus acumbens (a brain region that is part of the mesolimbic pathway whose core function concerns the cognitive processing of motor function related to reinforcement as well as in the regulation of slow-wave sleep) in teenagers tends to be greater in comparison to all other stages of life (24, 25).

An additional theory postulates that adolescents are hormonally pushed into biological maturity more quickly that they should be by societal standards, thereby creating dissonance between the teenager and society and societal rules (26). Accordingly, the teenager is autonomy-seeking to address this disparity (27).

In actuality, today we now know that the brain's gray and white matter obey different developmental expectancies (28). There are now those who postulate that risk-taking adolescents have structurally distinctive brains than those teenagers who are less prone to risk taking.

## Discussion

The prefrontal cortex serves as a brake on the limbic system but has a different developmental trajectory than that of the risk/reward brain region during the teenage years (29). The ability to relate functional as well as anatomical connectivities then becomes profoundly important in working with the police and departments of public health in reducing the increased risk of death and injury during the teenage years. The growth, development, and physiological as well as the social context must be taken into account when governments plan public health programming for adolescents. We have not done an adequate job in effecting the integration of such knowledge into youth programming and the referenced paper (30) is a welcome contribution to the investigation of factors that determine road traffic trauma among adolescents. We need to research other causes of trauma, falls at home, falls in public places and at work, the use of safety equipment at work and adherence to safe working protocols if we are to make progress in trauma prevention. Teenagers are risk prone and that affects mortality.

## Author Contributions

GL conceived and wrote the commentary. IW discussed and co-wrote the commentary. Both authors contributed to the article and approved the submitted version.

## Conflict of Interest

The authors declare that the research was conducted in the absence of any commercial or financial relationships that could be construed as a potential conflict of interest.
